# Effects of Tea Catechins on Alzheimer’s Disease: Recent Updates and Perspectives

**DOI:** 10.3390/molecules23092357

**Published:** 2018-09-14

**Authors:** Kazuki Ide, Norihiro Matsuoka, Hiroshi Yamada, Daisuke Furushima, Koji Kawakami

**Affiliations:** 1 Department of Pharmacoepidemiology, Graduate School of Medicine and Public Health, Kyoto University, Kyoto 606-8501, Japan; 2 Center for the Promotion of Interdisciplinary Education and Research, Kyoto University, Kyoto 606-8501, Japan; 3 Department of Drug Evaluation and Informatics, Graduate School of Pharmaceutical Sciences, University of Shizuoka, Shizuoka 422-8526, Japan; hyamada@u-shizuoka-ken.ac.jp (H.Y.); dfuru@u-shizuoka-ken.ac.jp (D.F.); 4 Jyoto Hospital, 11-22 Hanatenhigashi 2-chome, Tsurumi-ku Osaka-shi, Osaka 538-0044, Japan; kyotomatsu@hotmail.com

**Keywords:** Alzheimer’s disease, catechin, molecular mechanisms, clinical study

## Abstract

Alzheimer’s disease (AD) is one of the most common neurodegenerative disorders worldwide. Its incidence is gradually increasing because of an aging demographic. Therefore, AD prevention and modification is important to improve the health status of older adults. Oxidative stress is a component of the pathological mechanisms underlying AD. It is caused by a disruption of the balance between reactive oxygen species and antioxidant molecules. This imbalance also causes neuroinflammation. Catechins, which are bioactive components of tea, have antioxidative and anti-inflammatory effects. Moreover, other potential properties related to AD prevention and modification have been reported in in vitro and in vivo studies. Several clinical studies have also been conducted to date. The current review summarizes recent updates and perspectives of the effects of catechins on AD based on the molecular mechanisms and related clinical studies.

## 1. Introduction

Over 100 years ago, the first case of Alzheimer’s disease (AD) was reported by Dr. Alois Alzheimer, in a German woman, Auguste Deter. It was subsequently named “Alzheimer’s disease” by Dr. Emil Kraepelin and colleagues [1,2,3,4]. The number of individuals with AD is gradually increasing due to worldwide aging. The Alzheimer’s Association estimated the prevalence of AD in the U.S. in 2016 to be 5.3 million cases, and another study indicated that there were over 45 million individuals living with AD worldwide [5,6]. Two types of medications have been developed to treat AD symptoms: (1) acetylcholinesterase (AChE) inhibitors (donepezil, rivastigmine, and galantamine), and (2) an *N*-methyl-d-aspartate (NMDA) receptor antagonist (memantine). However, there is currently no cure [7]. In addition, the strategy for AD drug development has recently shifted toward disease prevention rather than treatment [7]. As such, a combination of pharmaceutical and nonpharmaceutical approaches is important.

Catechins, which are bioactive components of tea, have antioxidative and anti-inflammatory effects. Moreover, other potential properties related to AD prevention and modification have been reported in preclinical in vitro and in vivo studies of catechins [8,9]. Several clinical studies have also been conducted, and an intervention study is currently ongoing [10,11]. Hence, catechins and their derivatives have the potential be useful in pharmaceutical and nonpharmaceutical treatment approaches [11,12].

The current review introduces the pathophysiology of AD and summarizes recent updates and perspectives on the effects of catechins in AD based on what is known of the molecular mechanisms involved and research completed to date.

## 2. Pathophysiology of AD

AD is a progressive neurodegenerative disease and the most common cause of dementia (estimated to be responsible for approximately 60 to 80% of cases) [5]. Currently, amyloid and tau protein-related neurotoxicity, changes in cholinergic neurotransmission, oxidative stress, and alterations in calcium homeostasis are considered to be key elements of AD [13,14]. Amyloid proteins are processed from amyloid precursor protein (APP) by enzymes. When processed by β-site APP cleaving enzyme (BACE), amyloid β protein is produced, which triggers the development of AD [15]. Among various isoforms, 1-40 and 1-42 are most common, the latter of which is considered to exhibit the highest toxicity. The oligomerization and accumulation of amyloid β protein is associated with brain atrophy and cognitive decline [16,17]. Additionally, soluble amyloid β oligomers produce neurotoxic effects [18]. Therefore, balancing the generation and clearance of amyloid β is important during the development of AD pathology. Clearance is mediated by several receptors, including soluble and cell-surface lipoprotein receptor-related protein 1 (LRP-1) receptors [19]. A decrease in LRP-1 receptor expression and a corresponding increase in amyloid β production are observed in humans with age [20,21,22], and may underlie the age-associated accumulation of amyloid β in brain. Increased tau, a highly soluble protein related to microtubule structure and function, is also associated with AD pathology [23]. Microtubules are involved in neuronal growth and axonal transport. In AD, hyperphosphorylated tau protein is observed, leading to aberrant aggregation. This results in malfunctioning of axonal transport [24]. 

Increases in oxidative stress and neuronal inflammation are also related to neuronal dysfunction and neurodegeneration [25,26]. Oxidative stress is induced by an imbalance of reactive oxygen species (ROS) and antioxidants, which are increased in the brain with aging [27]. Mitochondria play an important role in maintaining the balance of ROS and antioxidants [28,29]. Neuronal inflammation can also be related to the pathogenesis of AD, and could be induced by the inflammatory reaction resulting from amyloid β protein accumulation [30]. In addition, systemic inflammation, obesity, and traumatic brain injury affect neuroinflammatory status [31]. Holmes and other researchers reported that systemic inflammation occurs prior to amyloid β deposition. Exacerbation of cognitive decline and behavioral changes after systemic inflammation have also been reported in humans [32,33]. Obesity itself increases bacterial and viral infections that cause systemic inflammation; furthermore, white adipose tissue secretes proinflammatory cytokines [34]. Traumatic brain injury not only directly affects learning and memory, but also decreases the degradation of amyloid β [35,36].

The main types of neurotransmitters involved in AD are cholinergic and glutamatergic transmitters [37,38,39]. The cerebral cholinergic system is thought to play an essential role in memory in humans [40]. Gil-Bea et al. reported that acetylcholine (ACh), cholinacetyltransferase (ChAT), and acetylcholinesterase (AChE) are all reduced in the frontal cortex of postmortem brains of individuals with AD [41]. Alterations in high-affinity choline uptake, impairment in ACh release, and other pathological changes in postmortem brains have also been reported and summarized elsewhere [42,43]. With respect to excitatory neurotransmission, glutamate in the synaptic cleft can be increased by the presence of soluble amyloid β oligomers [39], representing another target for AD drug development. Indeed, a glutamate receptor antagonist, memantine, is currently used to treat moderate to severe AD.

## 3. Molecular Mechanisms Underlying the Effects of Catechins in AD

**Figure 1:**
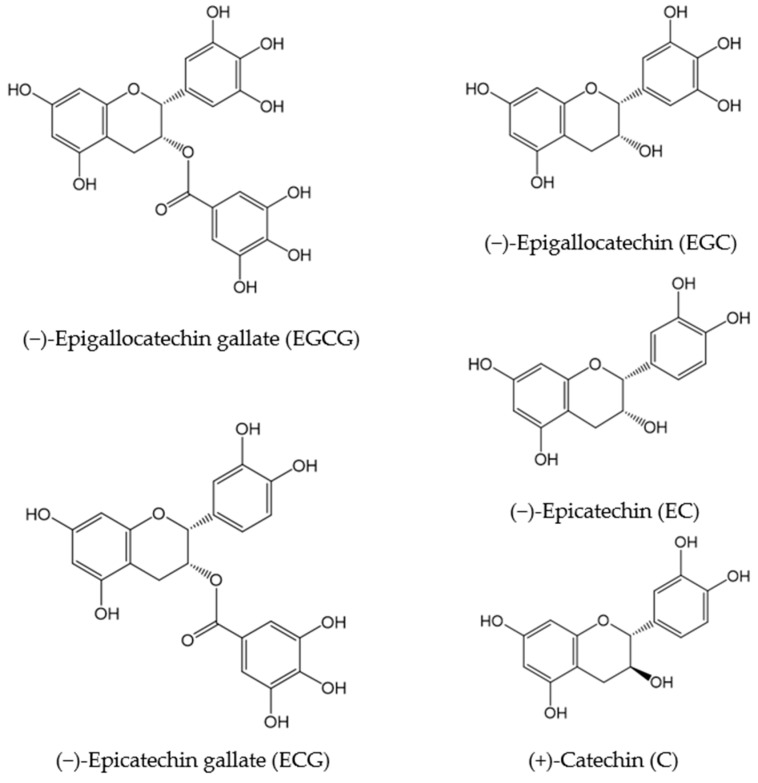
Chemical structures of several catechins.

A major bioactive compound constituting catechins is (−)-epigallocatechin gallate (EGCG) [8,44]. In addition, as reported by Lin et al., Japanese and Chinese green tea also contains (−)-epigallocatechin (EGC), (−)-epicatechin gallate (ECG), (−)-epicatechin (EC), and (+)-catechin (C) (Figure 1) [45]. These compounds are abundantly found in nonfermented teas, such as green tea.

Many studies on the molecular mechanisms of the effects of catechins on AD have been conducted in vivo and in vitro [46,47,48], as well as in silico [49]. Catechins’ known antioxidative effects may contribute to protection from neurodegeneration. As mentioned above, increased oxidative stress is involved in late-onset neurodegenerative disorders [50,51]. Indeed, peroxidized lipids, proteins, and oxidized DNA are known to be increased in individuals with AD [52]. Haque et al. reported that long-term (26 weeks) administration of green tea catechins (0.5% green tea catechins in water) prevented amyloid β-induced cognitive impairment in rats [53]. Along with preventing cognitive impairments, both hippocampal and plasma lipid peroxide and ROS levels were over 20% lower than controls, representing a significant reduction [53]. Biasibetti et al. also demonstrated a related effect of EGCG [54]. The authors evaluated the effects of EGCG using a streptozotocin-induced dementia model in the rat. After oral administration of 10 mg/kg/day of EGCG for a month, cognitive deficits assessed by the Morris water maze were reversed and ROS levels and NO production (based on nitrate in the hippocampus) were significantly reduced [54]. The radical scavenging activity of catechins [55] and metal iron chelating properties may contribute to these antioxidative effects [56,57]. Metal ions such as copper (II) and iron (III) can be chelated by catechins, and iron chelation reduces the production of ROS by inhibiting the Fenton reaction [58]. Copper (II) and iron (III) ions are also known to accumulate in the brains of individuals with AD [59]. These studies suggest that tea catechins can reduce oxidative stress in peripheral and brain tissue and that they may suppress behavioral changes related to cognitive deficits.

Catechins also possess anti-inflammatory properties, which may also underlie the mechanism of their effects on AD. Neuronal damage or injury leads to the secretion of proinflammatory elements (e.g., cytokines, cytotoxic elements), which trigger neuronal death [60]. In a study using lipopolysaccharide-injected mice (250 µg/kg/day for 1 week), Lee et al. demonstrated that preadministration of EGCG (1.5 and 3 mg/kg for 3 weeks) prevented lipopolysaccharide-induced memory impairment and suppressed the increase of cytokines and inflammatory proteins seen in nontreated controls [61]. Another in vitro study of BV-2 microglia showed that reactions related to lipopolysaccharide-induced inflammation (including nitric oxide production, cyclooxygenase-2 expression, and inducible nitric oxide synthase expression) were inhibited by EGCG [62].

Protein kinase C (PKC)-related mechanisms may also contribute to the effects of catechins on AD. PKC has a role in cell survival and soluble nontoxic amyloid β (sAPP) generation [63,64]. Several isozymes including α and ε activate α secretase, which leads directly to the cleavage of amyloid APP into nontoxic amyloid β. In vitro and in vivo studies published by Levites et al. revealed that a low concentration of EGCG (1–5 µM) stimulates sAPP production from human neuroblastoma and PC12 cells, and that 2 weeks of oral administration of EGCG (2 mg/kg/day) increases PKC α and ε in the hippocampus of mice compared to control-treated animals [65].

Other possible mechanisms of catechins have also been reported. Kaur et al. and Kim et al. conducted studies related to AChE inhibition using tea polyphenols [66,67]. In Kaur and colleagues′ study, aged Wister rats treated with green tea extract (0.5%) for 8 weeks showed significantly improved learning and memory performance, assessed using the passive avoidance test. AChE activity in the cerebrum was decreased in treated aged rats compared with young rats [66]. Kim et al. also reported similar results: amnesia induced by scopolamine was reversed by treatment with 0.2% (*w*/*w*) tea polyphenol administered to mice through the diet. Along with behavioral changes, AChE activity was strikingly inhibited by tea polyphenols [67]. In addition to these in vitro and in vivo studies, an in silico docking study with tea polyphenols and choline esterase enzymes has also been performed [49,68].

## 4. Clinical Studies on Catechins and AD

In 2018, over 10 clinical studies, including dose–response meta-analyses of observational studies on the effects of catechins on AD, were conducted in the United States, Europe, and Asia. Below, we briefly introduce examples of various study designs.

### 4.1. Cross-Sectional Studies

Since 2006, six cross-sectional studies have been conducted, the key information from which is summarized in Table 1. The first study was conducted in Japan by Kuriyama et al. [69]. The authors conducted a cohort study named the Tsurugaya Project in aged individuals, and epidemiologically analyzed the association between frequency of drinking green tea and cognitive function assessed by the Mini-Mental State Examination (MMSE). Among 1003 Japanese individuals aged >70 years, the prevalence of cognitive dysfunction (defined as the cutoff MMSE of 26) was lower in those who consumed more green tea (adjusted odds ratio (OR)) (95% confidence interval (95% CI)); ≤3 cups/week, 1.0 (references); 4 to 6 cups/week or 1 cup/day, 0.62 (0.33, 1.19); ≥2 cups/day, 0.46 (0.30, 0.72); *p* for trend <0.001) [69]. The results were consistent when the cutoff score of the MMSE was changed to 24 or 28. A study conducted on community-living Chinese aged ≥55 years also showed a negative association between green tea consumption and the prevalence of cognitive impairment (OR (95% CI) (reference: no consumption), 0.42 (0.25, 0.69)) [70]. Other studies reported by Nurk et al. in Norway and Feng et al. in Singapore also demonstrated similar associations [71,72]. Recently, Gu et al. reported the same trend in a Chinese population aged ≥60 years [73]. However, one study resulted in no significant association between green tea consumption and cognitive impairment [74], and another study showed sex differences within the effects [75]. In the latter study, the significant association was only observed in former and current green tea-consuming men, but not women, compared to controls [75].

### 4.2. Longitudinal Studies

Five longitudinal studies have been reported since 2008 (Table 1). The largest longitudinal study was conducted by Feng et al. in 2012 [76]. The study included over 7000 Chinese aged ≥80 years, and included a 7-year follow-up. Cognitive function, measured by verbal fluency tests, was higher at all time points in tea drinkers compared with nondrinkers. The positive correlation was also observed after adjusting for age, gender, years of education, and other background characteristics (regression coefficient, *p*-values; 0.72, *p* < 0.001 for daily drinking; 0.41, *p* = 0.01 for occasional drinking). 

In contrast to Feng′s study, which focused on an older population, Noguchi-Shinohara conducted a study named the Nakajima Project investigating individuals aged ~70 years at baseline [77]. The mean follow-up period (standard deviation) of the study was 4.9 (0.9) years. Among 723 individuals who participated in the study, 490 individuals completed the follow-up survey. Approximately 5% of individuals had an onset of dementia, and 13.1% of individuals were diagnosed with mild cognitive impairment (MCI) during the study period. When compared with non-tea-drinkers, the adjusted OR [95% CI] for cognitive decline in those who consumed green tea every day was 0.32 (0.16, 0.64). The association was also significant for those who consumed green tea 1 to 6 days/week. Interestingly, the associations were only observed using green tea, which contained a high content of EGCG, and was not observed using coffee or black tea as the variables of interest.

Three other longitudinal studies assessed the association between tea consumption and cognitive function. Ng et al. reported the study of community-living Chinese adults aged 65 years at the baseline [70]. In the study, 1438 participants reassessed the cognitive function by the MMSE at 1–2 years (median, 16 months) after the baseline assessment. A higher level of tea consumption was significantly associated with a lower prevalence of cognitive decline, even after adjusting for confounding variables. Arab et al. conducted a study in the United States, and the results suggested the existence of sex differences [78]. The authors followed up with participants of the Cardiovascular Health Study (CHS) for over 7 years, and an attenuated rate of cognitive decline measured by the MMSE was observed only in women. In addition, Eskelinen et al. reported no association between tea consumption and dementia/AD in an eastern Finland population [79]. However, as the authors discussed, the study only included a few individuals who drank tea. 

While observational results remain somewhat inconclusive, Liu et al. recently published the results of a pooled meta-analysis [80]. In total, data from 48,435 individuals were included, and tea consumption was found to be significantly negatively associated with the risk of cognitive dysfunction (OR (95% CI); 0.73 (0.65, 0.82) for any types of tea). When stratified by the type of tea, it was found that only green tea was responsible for this association (0.64 (0.53, 0.77)). The authors also evaluated the dose-dependent effects of green tea consumption, and a linear relationship was observed from 100 to 500 mL/day. Additional studies are needed to confirm this result; however, it is noteworthy that a meta-analysis showed a dose-dependent effect of tea consumption.

### 4.3. Interventional Studies

Several before–after and randomized-controlled studies also have been conducted to date (Table 2). One study used a green tea-based dietary supplement, and three studies used green tea itself. 

Park et al. reported on the effects of the supplement (LGNC-07), which included 1440 mg/day green tea extract [81]. Ninety-one individuals with mild cognitive impairment (MCI) participated in the study and took the supplement for 16 weeks. This study found no differences between supplement- and placebo control-treated participants with respect to memory and selective attention, although both were significantly improved among individuals who scored 21–23 on the Korean version of MMSE, regardless of treatment administered. 

All other studies using green tea were performed in Japan. In 2009, Kataoka et al. conducted a randomized study [82]. The authors used a capsule of tea powder and measured cognitive function using the Revised Hasegawa′s Dementia Scale (HDS-R). A 12-month intervention was performed, which demonstrated positive effects of theanine-rich green tea. However, the article fails to describe eligibility criteria, and the HDS-R was performed monthly and analyzed using multiple *t*-tests, a less robust method of analyzing repeated measures data. Therefore, the quality of the study is somewhat questionable. Following that, in 2014, our research group conducted a before–after exploratory intervention study [83]. Twelve individuals with cognitive dysfunction (defined as a score on the Japanese version of the MMSE (MMSD-J) of <28) aged ≥65 years (mean age 88 years) participated in the study and consumed green tea powder (2,000 mg/day; including 227 mg/day catechins). MMSE-J scores were significantly improved after 3 months of treatment (before: 15.3 ± 7.7; after: 17.0 ± 8.2; *p* = 0.03). Considering the results of this pilot study, we then conducted a double-blind, randomized-controlled study [84]. Thirty-three individuals participated, and 27 completed the study. Participants took 2000 mg/day of green tea powder or placebo powder for 12 months. Changes in the MMSE-J score during the study period were not significant between groups (least squares mean (95% CI); −0.61 (−2.97, 1.74); *p* = 0.59); however, levels of an oxidative stress marker (i.e., malondialdehyde-modified low-density lipoprotein (MDA-LDL) level) were significantly lower in the green tea group. The antibody for MDA-LDL is known to increase among individuals with AD [85]; therefore, the MDA-LDL-lowering effect of catechins may contribute to the suppression of AD progression. These results suggest potential effects of green tea on AD; additional large randomized-controlled studies are needed to confirm the effects.

## 5. Conclusions and Future Perspectives

The effects of catechins on AD have been investigated preclinically using various approaches (in vitro, in vivo, and in silico), and several clinical studies have also now been conducted.

As summarized in this article, as well as the functional effects of catechin treatment on cognition, experimental studies have revealed several putative mechanisms underlying the effects. Mechanisms related to antioxidative, anti-inflammatory, PKC-related, and neurotransmission-related properties of catechins may play a role. The structure–activity relationships between catechins and key enzymes involved in AD, revealed by an in silico docking study, also suggest potential mechanisms. A meta-analysis of clinical observational studies revealed a dose-dependent association after pooling the results of >45,000 individuals [80]. This dose-dependent association might support a robustness of effects in humans. However, the results of interventional studies are inconclusive. Three out of four studies demonstrated positive effects of tea and tea-based dietary supplement treatments; however, the sample size of these studies was relatively small (<100 individuals) and results of randomized studies were not consistent. This inconsistency might result from differences in study design and settings; additional research is needed to confirm the effects. Nonetheless, a study which demonstrated no improvement in cognitive function did reveal changes in a marker of oxidative stress, indicating that tea intake has some health benefit among individuals with cognitive dysfunction. Therefore, conducting additional studies is warranted.

Future studies should consider pharmacokinetic and pharmacodynamic aspects of catechins. Studies in rodents suggest that EGCG and other catechin compounds are blood brain barrier (BBB)-permeable, and a study using rat and human cell lines suggested a time-dependent course for catechins crossing the BBB [86,87,88,89]. For example, Nakagawa et al. reported that a single oral administration of 500 mg/kg EGCG reaches the brain at 0.5 nmol/g after 60 min [86], and another study demonstrated that 20 mg/kg of C and EC can permeate the blood brain barrier in rodents [88]. However, the bioavailability and distribution of catechins in the human brain remain to be elucidated. In addition to studies focused on the cognitive effects of treatments, clinical studies should also obtain basic physiological information. In this review article, we only focused on tea catechins, but grape (*Vitis vinifera*) seeds also include bioactive catechins such as EGCG. Therefore, additional studies that explore a wider variety of catechin sources are also important [90].

A combination of pharmacological and nutritional usage of catechins is also a possibility for future investigations. Chen et al. evaluated a combination therapy consisting of memantine and tea polyphenols [91]. In their study, the combination therapy more effectively protected from excitotoxic injury and impairment in locomotor activity than either component alone. Additionally, Zhang et al. [92] reported using a combination of catechins with acetylcholinesterase inhibitors. It is increasingly recognized that it may be important to focus on potential combinations of medical and nutritional approaches to establish the most effective future treatment options.

In summary, various studies have been conducted to date, and will continue to be performed, to examine the effects of catechins on AD. Several results suggest that catechins have promising therapeutic potential, which may contribute to improving the quality of life of aging populations.

## Figures and Tables

**Table 1:** Characteristics of cross-sectional and longitudinal studies.

Author, Year	Participants and Exposure	Results
*Cross-sectional studies*		
Kuriyama et al., 2006 [69]	Japanese residents ≥70 yo (*N* = 1,003); green tea	Prevalence of cognitive impairment was lower in the frequently tea-drinking group (aOR (95% CI) ^1^; ≥2 cups/day, 0.46 (0.30, 0.72); *p* for trend <0.001)
Ng et al., 2008 [70]	Community-living Chinese ≥55 yo (*N* = 2501); green, black, and oolong tea	Prevalence of cognitive impairment was lower in the frequently tea-drinking group (OR (95% CI) ^1^; medium-level intake, 0.45 (0.27, 0.720); high-level intake, 0.37 (0.14, 0.94))
Nurk et al., 2009 [71]	Norwegian residents 70–74 yo (*N* = 2031); any type of tea (black tea is most common)	Dose dependency for the cognitive performance was observed ~200 mL/day
Feng et al., 2010 [72]	Chinese residents ≥55 yo (*N* = 716); green, black, and oolong tea	Tea consumption was associated with improvement in cognition, executive function, and information processing speed (*p* < 0.01)
Gu et al., 2017 [73]	Participants of Weitang Geriatric Diseases Study ≥60 yo (*N* = 4579); any type of tea	Tea consumption was inversely associated with prevalence of cognitive impairment (OR (95% CI), 0.74 (0.57, 0.98), *p* = 0.032)
Shen et al., 2015 [74]	Chinese residents ≥55 yo (*N* = 9375); several types of tea ^2^	Compared with nonconsumption, tea consumption was significantly associated with lower prevalence of cognitive impairment (≥4 cups/day, OR (95% CI), 0.74 (0.57, 0.98), *p* = 0.032)
Huang et al., 2009 [75]	Chinese residents ≥90 yo (*N* = 681); any type of tea	In men, but not in women, lower prevalence of cognitive impairment among tea drinkers was observed (*p* = 0.044 for current drinkers)
*Longitudinal studies*		
Feng et al., 2012 [76]	Chinese residents 80–115 yo (*N* = 7139); any type of tea, 7-y follow-up	Cognitive function measured by verbal fluency tests was higher in all time-points for tea drinkers compared to nondrinkers. A positive correlation was observed after adjusting (regression coefficient, *p*-values; 0.72, *p* < 0.001 for daily drinking) ^3^
Noguchi-Shinohara et al., 2014 [77]	Japanese residents ≥60 yo (*N* = 723); green tea, 4.9-y follow-up	Compared with non-tea-drinkers, aOR (95% CI) for cognitive decline in those who consumed green tea every day was 0.32 (0.16, 0.64) (*p* < 0.05), and those who consumed green tea 1 to 6 days/week also showed a significant decline
Ng et al., 2008 [70]	Chinese residents ≥55 yo (*N* = 1438); green, black, and oolong tea; 1–2-y follow-up	High level of tea consumption associated with less decline in cognitive function (*p* = 0.042)
Arab et al., 2011 [78]	U.S. residents ≥65 yo (*N* = 4809); any type of tea; 7.9-y follow-up	An attenuated rate of cognitive decline, measured by the MMSE, was only observed in women (IRT-MMSE, *p* = 0.04)
Eskelinen et al., 2009 [79]	Eastern Finland residents 65–79 yo (*N* = 1409); 21-y follow-up	No association was observed between tea consumption and AD/dementia

^1^ Reference is the population of ≤3 cups/week; ^2^ green, black, oolong, pu-erh, scented, and fruit tea; ^3^ adjusting for age, gender, years of education, and other background characteristics. aOR, adjusted odds ratio; CI, confidence interval; IRT-MMSE, item response theory MMSE; OS, observational study; y, year; yo, years old.

**Table 2:** Characteristics of interventional studies.

Author, Year	Design, Participants, and Intervention	Results
Park et al., 2011 [81]	RCT, 40-75 yo with MCI, 16 weeks of LGNC-07 ^1^	Cognitive function and attention were significantly improved compared to controls (*p* < 0.05)
Kataoka et al., 2009 [82]	RCT, ≥85 yo with cognitive dysfunction, 12 months of theanine-rich green tea	Cognitive function measured by HDS-R was improved compared to control (*p* = 0.0306)
Ide et al., 2014 [83]	Before–after, ≥65 yo with cognitive dysfunction, green tea powder	Cognitive function measured by MMSE-J improved after the intervention (*p* < 0.05)
Ide et al., 2016 [84]	RCT, ≥50 yo with cognitive dysfunction, green tea/placebo powder	MMSE-J score was not significantly improved (LSM (95% CI); −0.61 (−2.97,1.74); *p* = 0.59); however, MDA-LDL level was lower in the green tea group (*p* = 0.04)

^1^ Tea-based dietary supplement; ^2^ Cognitive function measured by Rey–Kim memory test (*p* = 0.0478) and attention measured by Stroop test (*p* = 0.0306). HDS-R, revised Hasegawa’s Dementia Scale; MCI, mild cognitive impairment; LSM, least squares mean; MDA-LDL, malondialdehyde-modified low-density lipoprotein; MMSE-J, Japanese version of Mini-Mental State Examination; RCT, randomized controlled trial; yo, years old.
